# The agglomeration, coalescence and sliding of nanoparticles, leading to the rapid sintering of zirconia nanoceramics

**DOI:** 10.1038/s41598-017-02760-7

**Published:** 2017-05-31

**Authors:** Andraž Kocjan, Manca Logar, Zhijian Shen

**Affiliations:** 1 Department for Nanostructured Materials, Jožef Stefan Institute, Jamova 39, SI-1000 Ljubljana, Slovenia; 2 Department for Materials Chemistry, National Institute of Chemistry, Hajdrihova 19, SI-1001 Ljubljana, Slovenia; 3 Department of Materials and Environmental Chemistry, Stockholm University, Stockholm, Sweden

## Abstract

Conventional sintering is a time- and energy-consuming process used for the densification of consolidated particles facilitated by atomic diffusion at high temperatures. Nanoparticles, with their increased surface free energy, can promote sintering; however, size reduction also promotes agglomeration, so hampering particle packing and complete densification. Here we show how the ordered agglomeration of zirconia primary crystallites into secondary particle assemblies ensures their homogeneous packing, while also preserving the high surface energy to higher temperatures, increasing the sintering activity. When exposed to intense electromagnetic radiation, providing rapid heating, the assembled crystallites are subjected to further agglomeration, coalescence and sliding, leading to rapid densification in the absence of extensive diffusional processes, cancelling out the grain growth during the initial sintering stages and providing a zirconia nanoceramic in only 2 minutes at 1300 °C.

## Introduction

Sintering is the oldest method for the effective consolidation of particles into dense monoliths with tailored microstructures and properties. The studies setting the foundations date back to the early 1950s^1^. The conventional sintering of a porous green body consisting of coarse polycrystalline particles is a process of grain-growth-induced densification facilitated by atomic diffusion at approximately 2/3 of the melting temperature. It is thus a time- and energy-consuming process. During sintering the pores are annihilated, which leads to a sudden increase in the capillary forces that are created in the continuous pore network in order to reduce the surface free energy of the particles^2^. In order to promote the sintering activity, one option is to use nanoparticles, so that the increased surface free energy provides an enhanced driving force for sintering^3^. However, in practice, it is very difficult to densify nanocrystalline powders via conventional pressureless sintering techniques, because the nanoparticles are prone to agglomeration, which leads to inhomogeneous particle packing^4^. But if the agglomeration and extensive grain growth can be prevented^5^, pore-free nanoceramics with sub-100-nanometer grains^6^, having superior mechanical, optical, electrical, and/or thermal properties, can be expected^7^. An important step forward in achieving relatively dense nanoceramics was made by Chen and Wang^8^. By exploiting the differences in kinetics between grain-boundary diffusion and grain-boundary migration they discovered that it is possible to suppress the grain growth in the final stage of sintering. However, a moderate grain growth during the initial and intermediate stages of the sintering still resulted in at least a 4-fold increase in the grain size, leaving little room for manoeuvre when it comes to obtaining nanoceramics, especially for non-cubic materials^9, 10^.

We report on how, alternatively, primary crystallites can be used as the building blocks for homogenous packing and rapid sintering. Namely, the orderly agglomeration of 3 mol.% yttria-stabilized tetragonal zirconia (3YSZ) crystallites into secondary particle assemblies was beneficial for the more homogeneous packing of nanoparticles, while also preserving their high surface energy to higher temperatures, and as a consequence making it possible to sinter rapidly. When exposed to intense electromagnetic radiation, activated by a spark-plasma-sintering (SPS) set-up in a static graphite crucible, providing rapid heating at 250 °C/min, homogeneously assembled crystallites are subjected to further agglomeration, coalescence and sliding, cancelling out the moderate grain growth during the initial and intermediate stages of the sintering. In this way nanoceramics exhibiting 91% of theoretical density (TD) can be prepared at a heating rate of 250 °C/min and with only 2 minutes of dwell time at 1300 °C.

## Results and Discussion

**Figure 1:**
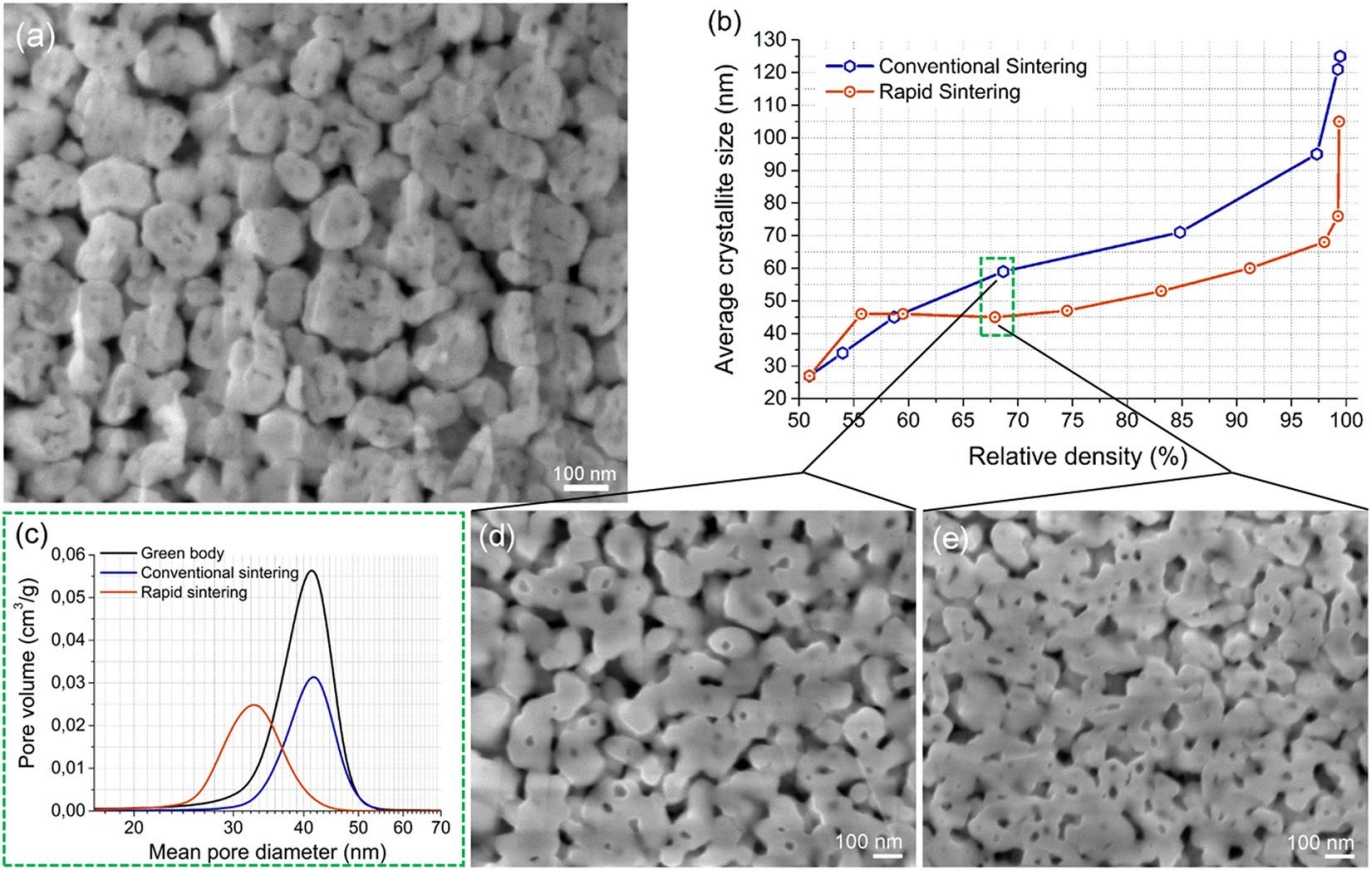
Observation of densification via crystallite growth and/or crystallite coalescence and rearrangement during conventional (CS) and rapid (RS) sintering. (**a**) 3YSZ green body with hierarchical heterogeneities originating from the dense and homogeneous packing of the loosely agglomerated nanocrystals forming secondary particles. (**b**) Crystallite size-density trajectories comparing the CS and RS of the 3YSZ green body derived from the Rietveld refined X-ray diffractograms. (**c**) Incremental pore size evolution during the RS and CS as determined by mercury porosimetry taken at the sintering conditions and relative densities from the green-dotted box in b and compared to the green body in a. (**d,e**) SEM images of the CS and RS samples sintered at 5 °C/min to 1100 °C for 2 h and 250 °C/min to 1200 °C for 2 min, respectively, to yield similar relative densities for comparison purposes.

We have previously shown that if the agglomeration of 3YSZ nanocrystallites is controlled in such a way as to form secondary particle assemblies with a narrow particle size distribution, a highly homogeneous packing of the nanoparticles can be ensured via a colloidal route, producing green bodies with hierarchical heterogeneities in terms of intra- and inter-particle pore packing^11^. Such microstructures (Fig. 1a) have the potential to prevent pore-boundary separation, as well as ensuring that the pores are smaller than the average particle size^6^, and simultaneously preserving the high surface free energy of the nanoparticles. At higher temperatures^12^, where their further agglomeration and coalescence can lead to rapid sintering, enhanced densification at increased heating rates with limited grain growth can be achieved. The rapid sintering (RS) of the 3YSZ green bodies was carried out in a pressure-less SPS set-up using a custom-made, static, graphite crucible (see Supplementary Fig. S1)^13^, which enables high heating rates, reaching the final sintering temperatures in several minutes. A heating rate of 250 °C/min was used to obtain final temperatures of 1100–1400 °C with 2 minutes of dwell. Such a sintering strategy employs an intense heat transfer from the graphite crucible to the compacts via electromagnetic radiation, while there is no externally applied pressure. For comparison, the same 3YSZ green bodies were sintered conventionally (CS) at a slow heating rate of 5 °C/min, with 2 hours of dwell time. A comparison of the crystallite size-density trajectories between the CS and RS strategies, made by Rietveld refinement of the corresponding X-ray diffraction (XRD) patterns (see Supplementary Fig. S2), is presented in Fig. 1b. The trajectory of the CS sample is in line with commonly observed trends during solid-state sintering, i.e., a more or less linear relationship, where the crystallite size increases with density^2^. The crystallite size exponentially increases after reaching a density of about 95%, where the grain-boundary-controlled diffusional processes dominate in the absence of the pore-pining effect due to the transition from a continuous pore network to isolated pores (closed porosity)^1^. In contrast, the trajectory in the RS sample exhibited two distinct features: i) a sudden jump in the crystallite size from 27 nm to 47 nm followed by ii) no notable growth throughout the densification up to about 70% TD, which roughly equals a dense arrangement of close-packed spheres. The observed features were indicative of intensive, agglomeration-driven densification via crystallite coalescence into larger single-crystalline entities, followed by their subsequent rearrangement during the RS. By comparing the pore size distributions (PSDs) for the CS and RS samples (Fig. 1c) taken under sintering conditions with similar densities of 68.7 and 67.9% TD, respectively (green-dotted box in Fig. 1b), it is clear that the mean pore diameter of the RS sample shifted to a smaller value (34 nm); in contrast to the CS specimen, where the median pore value (42 nm) remained unchanged. The “frozen” PSD is commonly observed during CS, where necks form between the grains in the initial and intermediate sintering stages^14^. On the other hand, the pore size decrease in the RS sample for a constant crystallite size suggests that agglomeration was taking place in order to reduce the surface free energy. SEM images of the ion-polished surfaces of the same specimens indicate a finer microstructure of smaller grains and pores for the RS sample. Moreover, the observed higher degree of interconnectivity of the grains, pore elongation, indicates a nanoparticle rearrangement.

**Figure 2:**
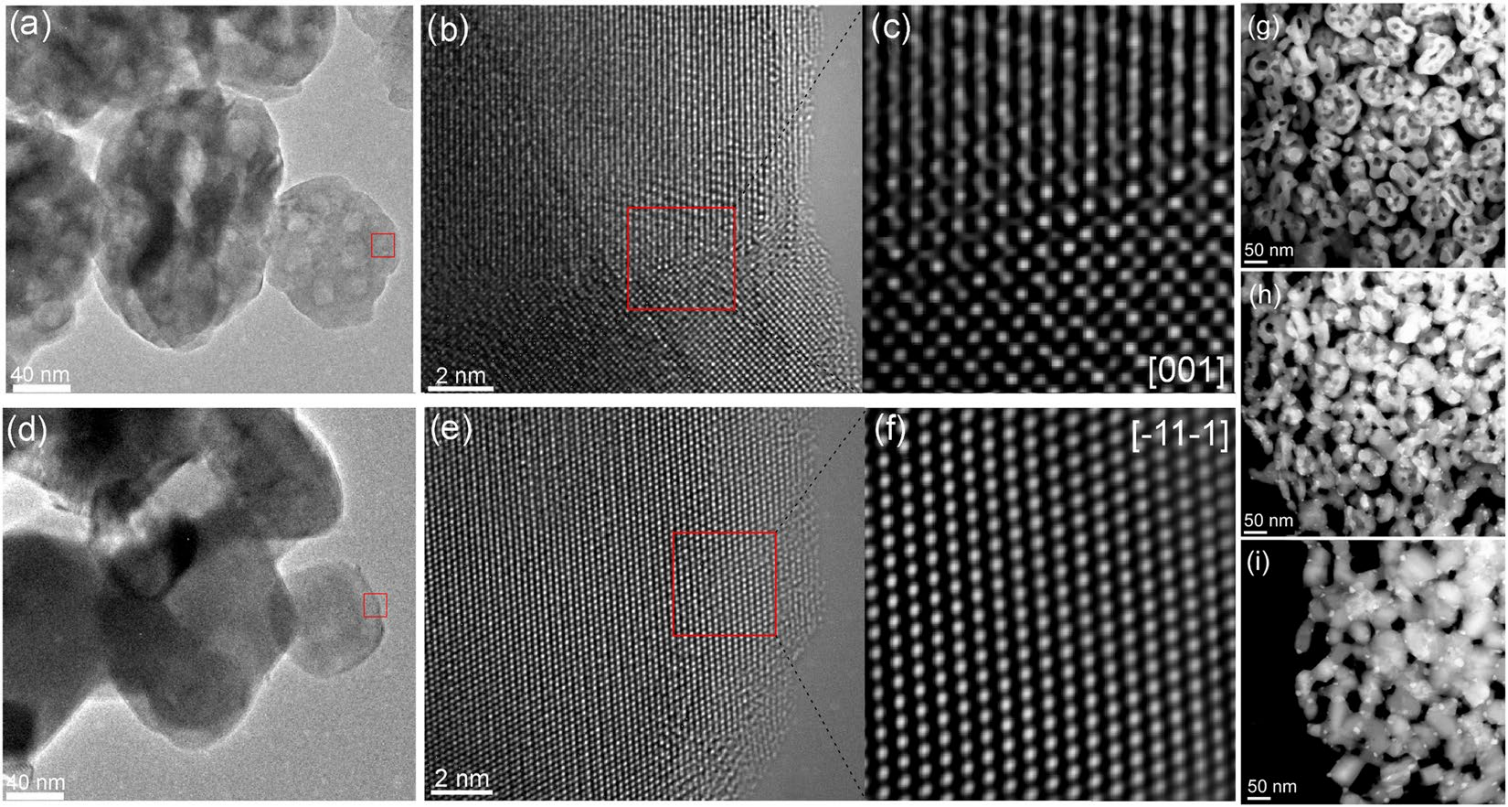
Observed 3YSZ crystallite coalescence within the secondary particle agglomerate into smaller, but denser, entities during the initial stage of rapid sintering (RS) in the TEM heating stage. (**a**) TEM image of the 3YSZ secondary particle agglomerate prior to RS. (**b**) High-magnification image of the area marked with a red rectangle from a and, (**c**) HRTEM image showing a boundary between two neighbouring nanocrystals forming the secondary mesoporous particle. (**d**) TEM image of the same site as in a after RS at 250 °C/min to 1200 °C with no dwell time. (**e**) High-magnification image of the rectangle from d indicating the absence of crystallite boundaries and, (**f**) HRTEM image showing single-crystalline nature of the initially polycrystalline, secondary 3YSZ nanoparticle from a after RS. TEM dark-field micrographs of 3YSZ bulk body intersecting the heating process at (**g**) 600 °C, (**h**) 1100 °C, and (**i**) 1200 °C.

The initial sudden jump in crystallite size at the on-set of the RS process indicates a coalescence of the primary crystallites. *In-situ* TEM heating experiments enable mimicking of the sintering processes and monitor the coalescence of oxide nanoparticles at elevated temperatures^15–17^. However, it is expected that the temperatures at which the changes take place will differ and cannot be directly compared due to the difference in volume of the sample to be heated and the system employed. Figure 2 presents the results from the TEM heating stage and the HRTEM analyses obtained when heating the employed 3YSZ mesoporous powder. Figure 2a shows several 3YSZ secondary particle agglomerates prior to heating, composed of loosely aggregated crystallites with an average crystallite size of 27 nm^11^. In the bottom-right-hand corner of the image a smaller, about 100-nm, agglomerate resides, where the spheres of light-greyish colour are indicative of the presence of mesopores as a result of the loose packing of the primary crystallites. In Fig. 2b, a high-magnification image of the red rectangle shows a crystallite boundary between two neighbouring crystallites forming the secondary particle. The crystallite boundary is clearly discernible from the HRTEM image (Fig. 2c). In order to mimic the RS process the sample was rapidly heated *in-situ* in the TEM at 250 °C/min to 1200 °C. No changes were observed up to 900 °C (see Supplementary Fig. S3a). Within a timeframe of 48 seconds at 1100 °C, parts of the observed agglomerate (Fig. 2a) became darker, displaying the thickness inhomogeneities, the outer particle rim had several round-shaped bumps, and the light-greyish mesopores were smaller in size (see Supplementary Fig. S3b). After an additional 19 seconds of heating at 1180 °C, the thickness inhomogeneities were removed together with the complete disappearance of the mesopores (see Supplementary Fig. S3c). The observed agglomerate shrank by 20% in diameter (Fig. 2d) and was converted into a monocrystalline nanoparticle, since the inner-crystallite boundary disappeared (Fig. 2e,f). The indexing of the crystalline planes and zone axes of the HRTEM images (Fig. 2c and f) were made (see Supplementary Fig. S4). The observed nanoparticle shrinkage and the disappearance of the crystallite boundary (Fig. 2) can explain the 27–47 nm sudden initial jump in the crystallite size (Fig. 1a). The observed features are direct evidence of the crystallite coalescence and the pore shrinkage, which both contributed to the initial, diffusionless densification of the 3YSZ secondary mesoporous particles in the initial stage of rapid sintering during rapid heating at temperatures lower than one-half of the melting temperature.

The same processes were also recorded with dark-field TEM in a thinned, 3YSZ, green body prepared for *in-situ* TEM analysis (Fig. 2g–i). The mesopores were almost entirely annihilated during the heating from 600 °C (Fig. 2g) to 1100 °C (Fig. 2h) at 250 °C/min, while at 1200 °C they were completely removed (Fig. 2i). Moreover, the close proximity of very small, randomly oriented, crystallite domains was observed within a primary crystallite (see Supplementary Fig. S5a,b), which can contribute to a sudden increase in the crystallite size, being similar to the oriented-attachment (OA) mechanism operating in colloidal aqueous media^18–20^. In the OA mechanism the primary crystallites combine with their high-energy interfaces, thereby releasing their free surface energy. Besides, it was shown for tin oxide that the rotational movement of the particles with boundaries was triggered by heating, even if the contact points and/or contact faces with neighbours was restricting their mobility prior to heating^17^. However, the driving force for the surface-free-energy minimization of the nanoparticles during rapid heating, inducing their coalescence, is still not fully understood. Previously, it was shown that applying electric fields in the absence of any current can accelerate the sintering processes, apparently as a result of the lowered activation energy for point-defect formation^21–23^, the dielectric breakdown of insulating surface layers^24, 25^, and interactions with space-charge layers^26^.

The TEM heating stage was further utilized to follow the densification within the intermediate stage of sintering, which exhibited no further notable crystallite growth (Fig. 1a). Such intensive, agglomeration-driven, enhanced densification was directly observed when the 3YSZ secondary particle agglomerates (or grains) were placed on the TEM grid and were rapidly heated at 250 °C/min to 1200 °C. Several micrographs were taken at different dwell times (see Supplementary Fig. S6a–d). In a time frame of only 6 minutes of dwell there were obvious indications of grain sliding and agglomeration consisting of several 3YSZ grains that had been coalesced into larger monocrystalline grains, while the pores shrank and were annihilated as a result of the densification process (Fig. 1c). It was recently shown that the particle coalescence and the pore shrinkage of 3YSZ agglomerates were triggered by an electric field, but in the absence of any current, resulting in a 7% agglomerate shrinkage at 900 °C. Apparently, the applied electrostatic potential lowers the activation energy for point-defect formation within the space charge zone and, hence, promotes neck formation and consolidation^21^.

**Figure 3:**
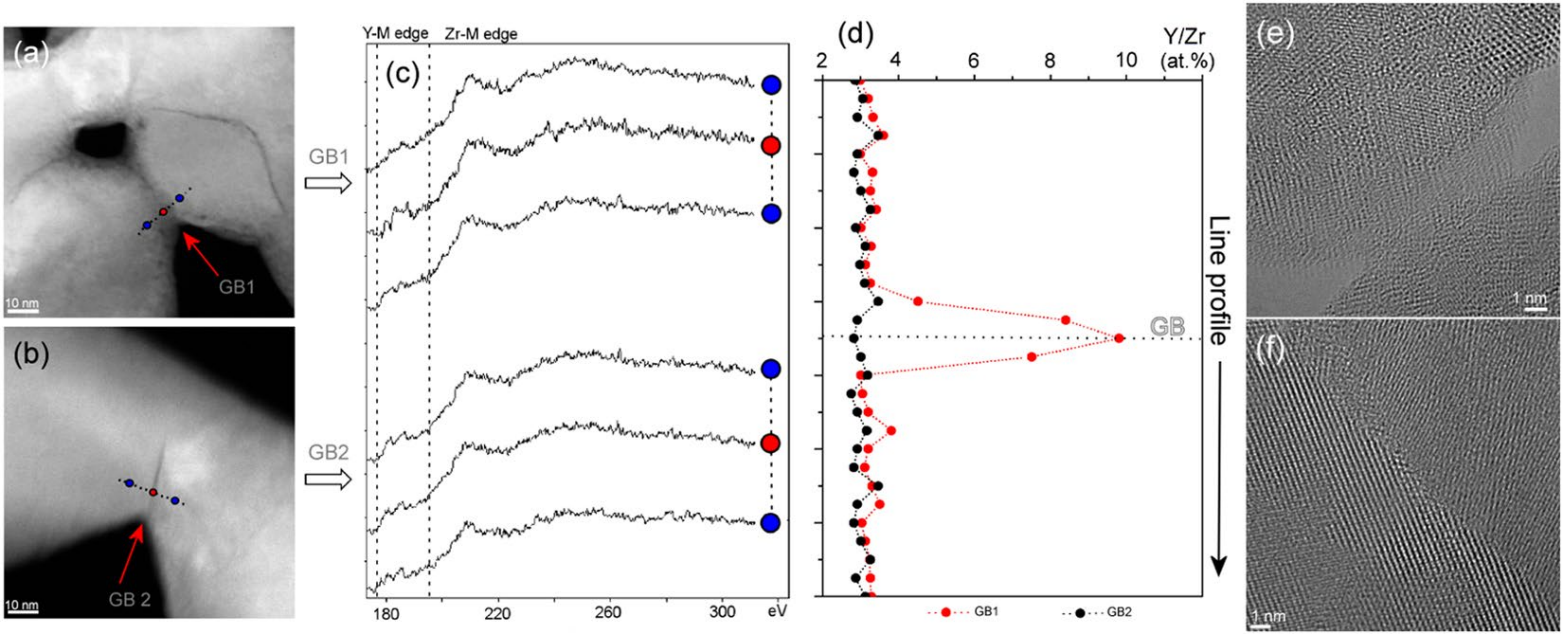
STEM-HAADF, EELs and HRTEM analyses of the crystallite boundaries of the 3YSZ green body sintered by the rapid (RS; 250 °C/min, 1100 °C, 2 min) and conventional (CS; 5 °C/min, 1050 °C, 2 h) sintering processes yielding similar densities of approximately 58% of theoretical density. STEM-HAADF images of (**a**) RS and (**b**) CS showing their crystallite boundaries. (**c**) EELS spectra acquisition of Y-M and Zr-M was performed in a line profile across the crystallite boundary depicted on a and b. (**d**) Concentration profile of Y/Zr (at.%) across the crystallite boundary depicted on a and b for RS and CS samples, indicating the presence of a thin, yttria-rich, amorphous, liquid-like, intergranular film (yttria segregation) located at crystallite boundaries in the RS sample. HRTEM micrographs displaying crystallite boundaries of (**e**) the RS sample showing the presence of an amorphous layer (IGF-like film) and of (**f**) CS sample from b showing a clear, fully crystalline boundary.

In our case, the intensive, agglomeration-driven, enhanced densification on account of the grain sliding and rearrangement, with the absence of crystallite growth, during RS employing intense electromagnetic radiation was striking, reaching a density (Fig. 1a) between randomly packed, uniform spheres in a close-packed system. The extent of such sliding and rearrangement was only found in liquid-phase sintering systems as an early-stage densification mechanism. It occurs in response to the capillary force, an equivalent hydrostatic pressure, arising from the curvature of the liquid meniscus between neighbouring grains, providing the sintering driving force^27, 28^. The STEM-HAADF technique (Fig. 3) was used to analyse the grain boundaries in order to gain more information about their state just after the initiation of the intermediate stage of sintering via the RS and CS strategies, reaching 55.7 and 58.7% TD, respectively, which were obtained, however, under specific sintering conditions encountered during each sintering strategy. Differences can be observed from the STEM micrographs (Fig. 3a,b). In the RS case the grains have a core-shell structure, where their edges and boundaries are darker (Fig. 3a), but not such a dark contrast as in the case of CS (Fig. 3b). Bright-field HRTEM analyses confirmed the differences in terms of the degree of crystallinity of the boundaries (Fig. 3e,f). In the RS sample an amorphous-like intergranular film (IGF) can be observed, while in the case of CS the grain boundary is completely crystalline. The quantification of elements obtained by the EELS across the grain boundaries (Fig. 3c) revealed a higher content of yttrium within the amorphous region of the boundary of the RS sample, while there were no changes in the yttrium concentration across the examined region in the case of the CS sample (Fig. 3d). It is likely that the IGF originates from the initial 3YSZ powder as a result of the synthesis conditions. The loosely aggregated primary crystallites consisting of 3YSZ powder were prepared by a hydrothermal process in which the inhomogeneous distribution and segregation of yttria on the zirconia nanoparticles’ surfaces results from the hydrolysis kinetics difference of the respective cations. It follows that the segregated yttria can be homogeneously incorporated into the lattice of tetragonal zirconia nanoparticles during the slow process of CS, whereas during RS such inhomogeneities are preserved, even to higher temperatures, due to the lack of time for atomic diffusion to take place. Moreover, the yttria-rich IGF can potentially influence the local ionic conductivity within the grain boundaries and, thus, the dielectric breakdown, yielding local melting due to a locally enhanced electromagnetic field between the particles, preserving the existence of the IGF even more. Such an amorphous-like IGF can be regarded as a thin layer of liquid allowing the activation of the increased sintering, hydrostatic pressure acting on the necks of the distorted grain boundaries, leading to intensive, agglomeration-driven, enhanced densification as a result of the grain sliding and rearrangement with no grain growth during the intermediate stage of sintering. A simplified calculation of the initial sintering pressure yields values exceeding 100 MPa, assuming 100-nm secondary particles, i.e., grains, which can be more than one order of magnitude higher than the typical pressures encountered with micron-sized powders^27^, representing a considerable force, high enough to induce rearrangement to the extent observed in the present study.

**Figure 4:**
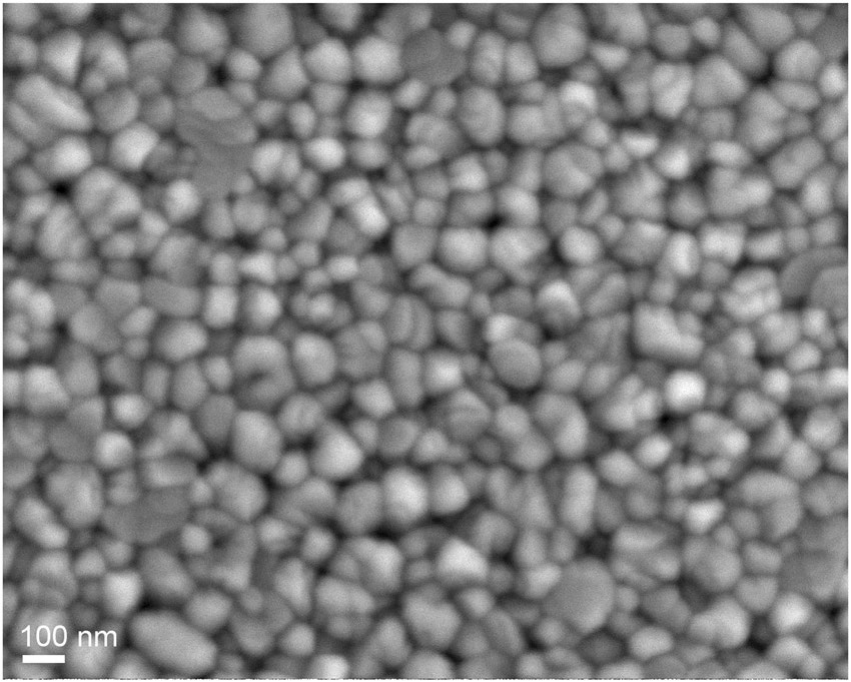
SEM image of 3YSZ nanoceramics. Rapid sintering at a heating rate of 250 °C/min to 1300 °C with 2 minutes of dwell time was employed. The density was 91% TD, the average grain and crystallite sizes were 106 nm and 60 nm, respectively.

The result of the RS of 3YSZ primary crystallites being agglomerated into the secondary mesoporous particles, i.e., grains, is the possibility to prepare nanoceramics (Fig. 4). By heating rapidly at 250 °C/min to 1300 °C with 2 minutes of dwell, the nanoceramics exceed 91% TD, having an average crystallite and grain size of 60 and 106 nm, respectively. The majority of the individual grains are polycrystalline and composed of smaller domains as a consequence of the grain sliding and rearrangement. The microstructure gives the impression of a fully dense body, although some mesopores between the grains are visible, indicating the lack of time for extensive surface-diffusion processes, which, if present, would round the pores and merge them into larger entities in order to lower their surface energy^6, 29, 30^. Even if not completely dense, the RS 3YSZ nanoceramics possess interesting mechanical and thermal properties. The bending strength exceeded 600 MPa, the while the Vickers hardness and indentation toughness were 9.3 GPa and 4.8 MPa·m^1/2^, respectively. Due to the amount of mesoporosity and the nanoscale crystallites the thermal conductivity was reduced by almost 1/3, i.e., from 2.8 to 2.0 W/mK, when compared to a dense standard.

In summary, we have shown that primary crystallites can be used as building blocks for homogenous packing and rapid sintering. The orderly agglomeration of 3YSZ crystallites to form secondary nanoparticle assemblies can be beneficial for the more homogeneous packing of nanoparticles to form green bodies, while also preserving their high surface energy to higher temperatures. When exposed to intense electromagnetic radiation, activated by the SPS set-up in a static graphite crucible, providing rapid and effective heating at 250 °C/min, the homogeneously assembled crystallites further agglomerate, coalesce and slide, leading to rapid densification in the absence of extensive diffusional processes, cancelling out the moderate grain growth during the intermediate stage of sintering that was followed by an initial primary crystallite coalescence during the on-set of rapid heating. Such an agglomeration-driven, enhanced densification of the nanoparticles was facilitated by the presence of a thin, yttria-rich, amorphous, liquid-like, IGF located at the grain boundaries, which allowed the activation of an increased capillary, hydrostatic sintering pressure, with which the 3YSZ nanoparticles were densified to 91% TD at 1300 °C with only 2 minutes of dwell time and with minimal growth due to the absence of extensive diffusion processes. The result was 3YSZ nanoceramics. The presented rapid-sintering strategy has the potential to be a cost-effective, pressureless sintering process for nanoceramics with improved properties. On the other hand, the observed mechanisms of crystallite coalescence and rearrangement during the rapid heating could shed more light on the densification and grain-growth mechanisms operating in other rapid-sintering methods. Finally, this finding could turn the disadvantage of agglomerating nanocrystals into a favourable one for preparing bulk nanomaterials.

## Methods

### Nanocrystalline 3YSZ mesoporous powder

In the present study a custom-made nanocrystalline 3YSZ mesoporous powder with 2.94 mol.% of yttria and a small amount of alumina (0.2–0.4 wt%) was used. The powder is a semi-product with the potential to be commercialized in the near future, and so the producer will not be disclosed. It consists of 100–150-nm, spherically shaped particles. The single, secondary particle is composed of loosely aggregated, primary, nanoscale crystallites. The crystallites’ average size was estimated to be 27 nm, according to a Rietveld refinement of the XRD pattern. The specific surface area of the powder was 18.2 m^2^/g, as measured by the BET. A BJH pore-size analysis revealed that it consists of intra-particle pores with an average size of about 8 nm.

### Sample preparation for sintering

To achieve an enhanced particle packing, a colloidal route for sample preparation was employed by the centrifuge casting of weakly flocculated suspensions in order to avoid particle segregation. The suspensions were prepared by dispersing 3YSZ mesoporous powder in water to achieve 35 vol.% of solids loading, where citric acid was used as a dispersant (0.45 mg/m^2^) and ammonium hydroxide for adjusting the pH value of the suspension to 8. The suspension was homogenized by ball milling overnight in a plastic container using zirconia balls with an average diameter of 5 mm. The suspension was then diluted to 30 vol.% by adding an appropriate amount of tribasic ammonium citrate salt to achieve a 0.5 mol/l concentration, by which weak flocculation of the suspension was induced. The suspension was then mixed with a magnetic stirrer for 2 hours. The slip casting of the 3YSZ powder aqueous suspensions was performed in a centrifuge (Megafuge 1.0, Heraeus Instruments GmbH, Hanau, Germany) at 4000 rpm for 30 min in a specially designed plastic mould (φ = 20 mm), which roughly corresponded to a centrifugal force of 2860 g. Before casting the moulds were sprayed with a Teflon-based dry lube so as to increase the hydrophobicity and to prevent sticking of the specimens after drying. Drying was performed in a humidity chamber overnight at 35 °C and 80% of relative humidity. Afterwards, the specimens were heat treated in a chamber oven in order to remove the organics with the following regime: 2 °C/min to 600 °C with a dwell time of 2 hours.

### Sintering

The sintering was performed in an SPS furnace (SPS-615E, Dr. SINTER, SPS Syntex Inc., Japan) under vacuum conditions. A custom-made graphite crucible was fabricated for the sintering experiments with an inner volume of approximately 12 cm^3^ by passing a pulsed, direct, electrical current through the crucible. Samples were placed inside it on the 1-cm-thick graphite wool to prevent contact with the bottom of the crucible. The temperature was measured with a pyrometer by pointing it on the crucible wall with a drilled hole of 2 mm. The final sintering temperatures of the SPS were in the range1100–1400 °C, the dwell time being 2 min, and a heating rate of 250 °C/min. After the sintering process was completed the samples were furnace cooled. The samples were slightly greyish in colour, but after re-oxidation in a chamber oven in air at 950 °C for 4 hours they became completely white. For comparison, the same 3YSZ green bodies were sintered conventionally (CS) in a resistance chamber oven in ambient air at a slow heating rate of 5 °C/min, with final temperatures of 1000–1350 °C with 2 hours of dwell.

### Characterization

Microstructural characterization of the green and sintered samples was performed with a Jeol JSM-7000F SEM (Jeol, Tokyo, Japan) at an accelerating voltage of 5 kV on ion-beam polished surfaces prepared using an SM09010-CP ion-beam polisher (Jeol, Tokyo, Japan), where argon was used as a source of ions at 5 kV. The sintered samples from Fig. 4 was polished and thermally etched at 1200 °C for 3 minutes.

The pore volume and the pore size distribution of the porous monoliths were determined using an AutoPore III 9410 mercury-intrusion porosimeter (Micromeritics, Norcross, GA, USA) within the pore-diameter interval 10 nm to 360 µm. The surface tension and the contact angle of the mercury were set to 0.485 mN/m and 130°, respectively. The densities of the specimens were estimated either by using mercury-intrusion porosimetry or by Archimedes’ method, with water as the immersing medium.

XRD patterns of the as-received powder and partially sintered samples were recorded using a PANalytical X’pert PRO MPD diffractometer (PANalytical, Almelo, Netherlands) equipped with a PIXcel detector and using a Cu–Kα1 radiation source. The measurements were carried out using fixed divergence (1°) and anti-scattering slits, a 10-mm mask, a continuous scan mode in the 2θ range 20–100°, with a step size of 0.00656° and a scan speed of 0.01113°/s. The crystallite size analysis from the X-ray powder diffraction peak broadenings was performed with the Rietveld method using the program FullProof (FullProof Suite).

The grain size was evaluated using the linear-intercept method with no correction factor employed.

A FEI Titan transmission electron microscope (TEM) operating at 300 KV was used for imaging and additional composition/phase analysis. For the *in-situ* imaging an Aduro holder from Protochips was employed in the experiments. EELs acquisition was performed in a FEI Titan Scanning Transmission Electron Microscope (STEM) operating at 200 eV. The EEL spectra were acquired using the line-profile feature across the grain boundary with Titan Imaging and Analysis (TIA) software. During the experiment, the incident electron beam was perpendicular to the grain boundary. Each spectrum was collected every 1 nm. The HAADF image was also taken during the scan. Before and after each scan, a STEM bright-field image was taken to check that the specimen’s damage and carbon contamination did not occur during the spectra acquisition. The EELs spectra were collected with 1.0 ± 0.2 eV at a dispersion of 0.2 eV per channel, a convergence semi-angle of 9.6 mrad and a collection semi-angle of 24 mrad. The spectrum processing for elemental quantification was performed with Digital Micrograph EELS analysis. Fourier-log deconvolution processing was employed to remove the plural scattering. The background of the spectra was subtracted using the power-law model. The quantification of the yttrium (Y) and zirconium (Zr) elements at the grain boundary was calculated from the EELS core-loss region. The dopant concentrations were obtained from a Hatree-Slater calculation of the ionization cross-sections for the zirconium and yttrium L edges.

## Electronic supplementary material

Supplementary information
