# Supplementary material for: The agglomeration, coalescence and sliding of nanoparticles, leading to the rapid sintering of zirconia nanoceramics
Source: Sci Rep. 2017 May 31;7:2541. doi: 10.1038/s41598-017-02760-7 (PMC5451455; doi:10.1038/s41598-017-02760-7)
Supplement: Supplementary file 1 — Supplementary information [file 41598_2017_2760_MOESM1_ESM.pdf]

Supplementary Information

The agglomeration, coalescence and sliding of nanoparticles, leading to the rapid sintering of zirconia nanoceramics

Andraž Kocjan, Manca Logar, Zhijian Shen*

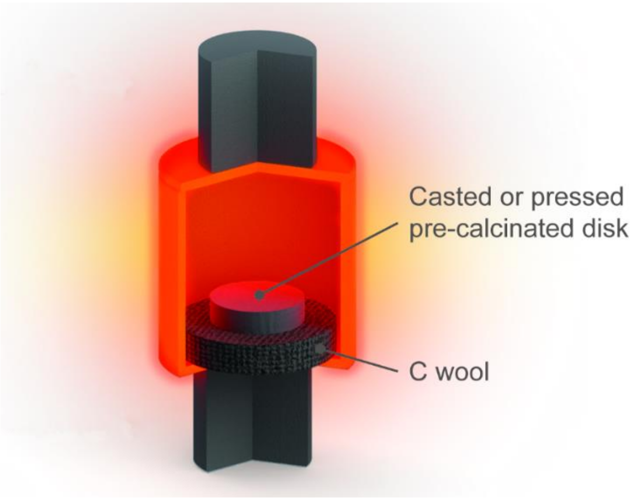

Figure S1. Scheme of a custom-made static graphite crucible used with SPS for the rapid sintering of 3YSZ samples.

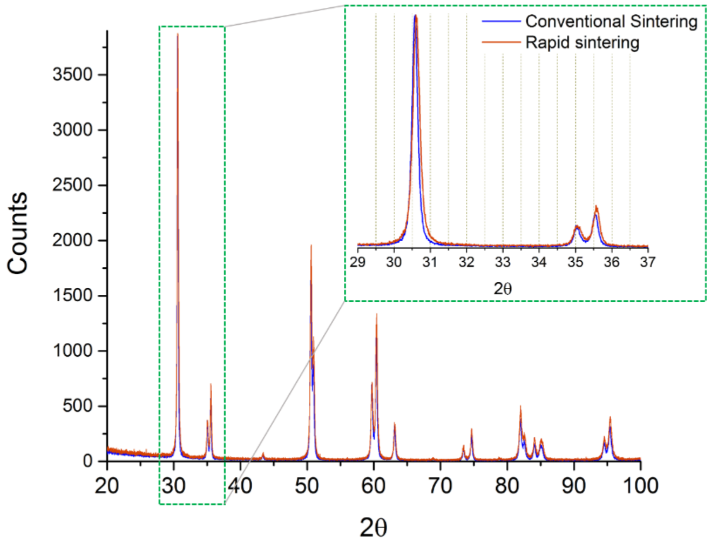

Figure S2. Representative pair of XRD diffractograms for CS and RS samples displayed in Figure 1c-e. Both samples exhibit a tetragonal zirconia phase

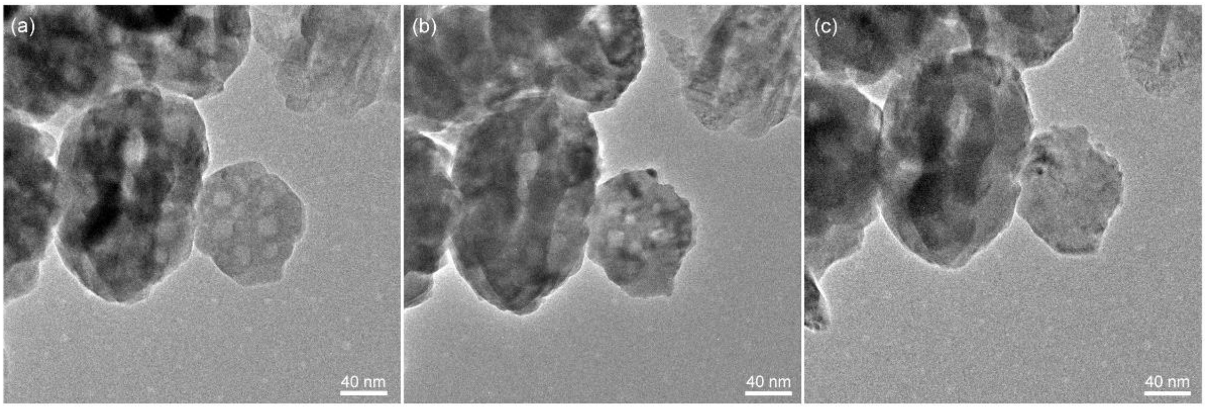

Figure S3. Densification of the zirconia secondary particles via direct crystallite migration and coalescence during rapid heating at 250 °C/min. TEM micrographs were taken when the temperature of the heating stage reached a) 900 °C, b) 1100 °C, and c) 1180 °C.

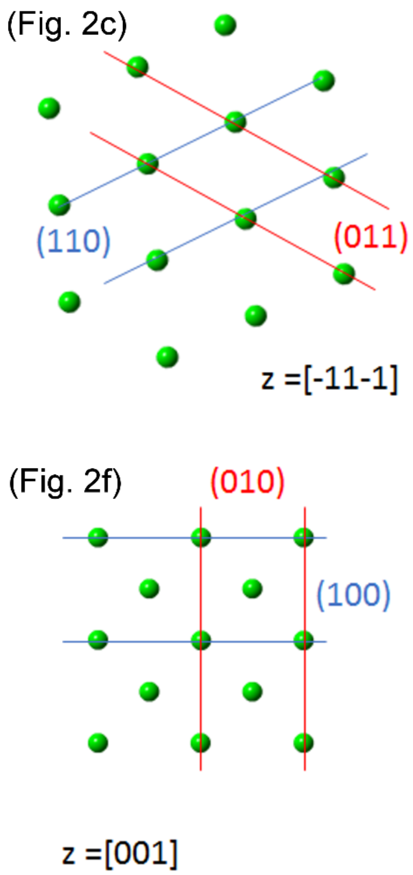

Figure S4. Indexing of the crystalline planes and zone axes corresponding to the HRTEM images shown in Figure 2c and 2f.

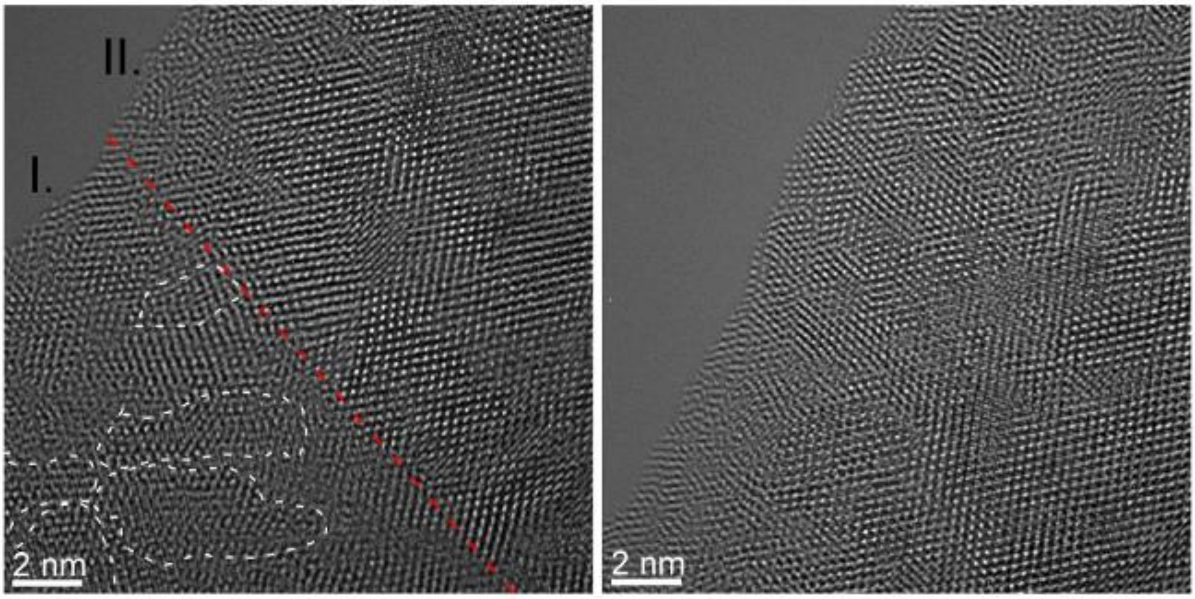

Figure S5. a) The close proximity of several randomly oriented, nanoscale, inner-crystallite domains observed within the single 3YSZ primary crystallite composing a secondary particle in the TEM heating stage prior to rapid heating and b) their reorientation and alignment into the orientation of the hosting crystallite, forming a single-crystalline structure after rapid heating at 250°C/min to 1200°C.

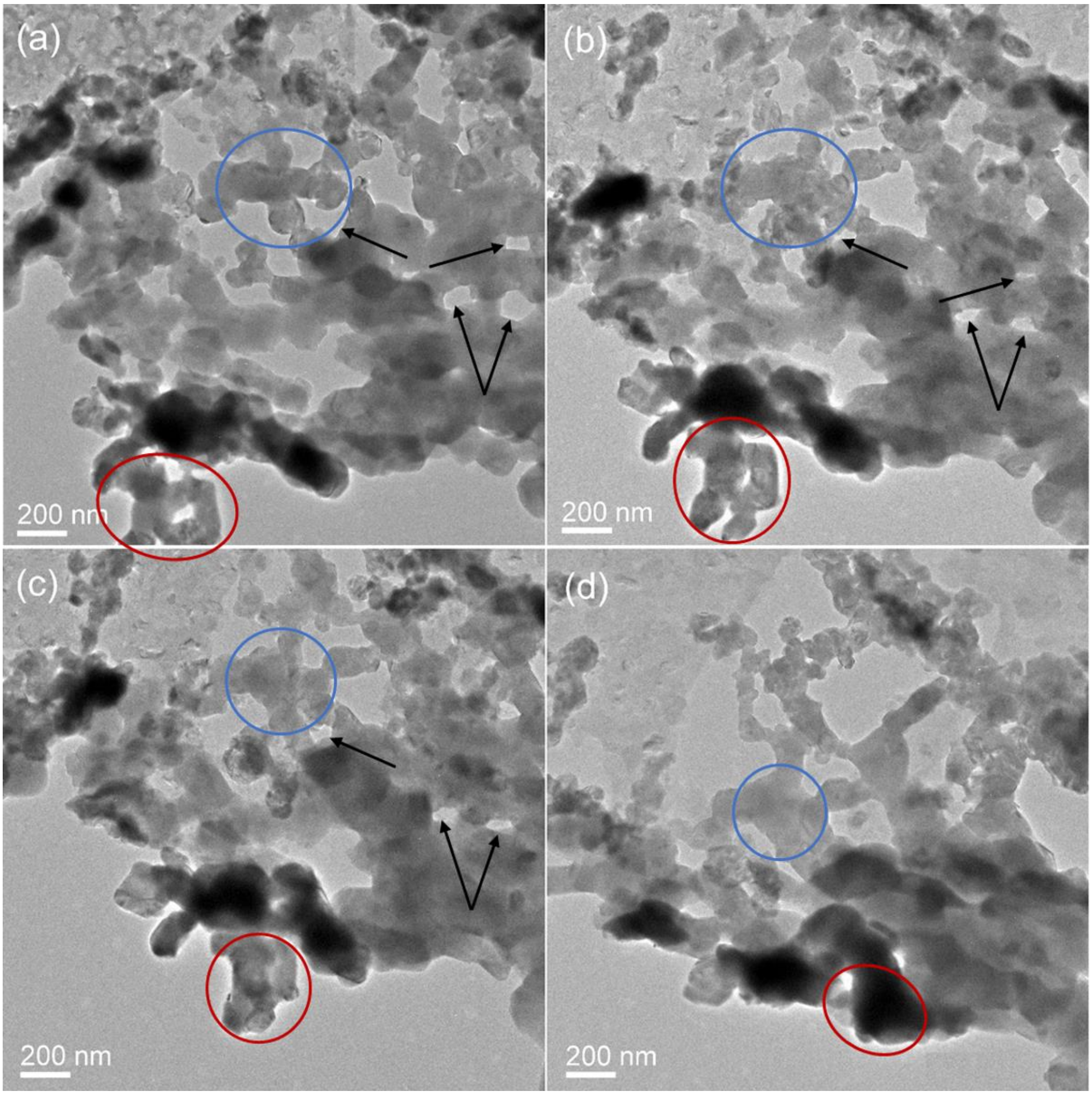

Figure S6. a) Densification process of the 3YSZ powder placed in the TEM heating stage during rapid heating at 250°C/min to 1200°C and dwell times of a) 0 minutes, b) 3 minutes, c) 4 minutes, and d) 6 minutes. Colour circles are indicating the same positions, where nanocrystal agglomeration, sliding and coalescence can be observed. Arrows indicate the pores that were annihilated after only 6 minutes of dwell time.
